# The needs and service preferences of caregivers of youth with mental health and/or addictions concerns

**DOI:** 10.1186/s12888-020-02801-y

**Published:** 2020-08-14

**Authors:** Roula Markoulakis, Samantha Chan, Anthony Levitt

**Affiliations:** 1 Family Navigation Project, Sunnybrook Research Institute, Toronto, Ontario Canada; 2 Department of Occupational Science and Occupational Therapy, Faculty of Medicine, University of Toronto, Toronto, Ontario Canada; 3 Hurvitz Brain Sciences Program and Department of Psychiatry, Sunnybrook Health Sciences Centre, Toronto, Ontario Canada; 4 Department of Psychiatry, Faculty of Medicine, University of Toronto, Toronto, Ontario Canada

**Keywords:** Navigation, Youth, Mental health, Addictions, Caregivers, Health services

## Abstract

**Background:**

Caregivers experience significant strains as a result of navigating the complex mental health and/or addiction (MHA) system for their youth with MHA issues. We examined the characteristics of Ontario families with youth with MHA issues and their service needs.

**Methods:**

A cross-sectional survey study investigated the characteristics and service needs of families with youth with MHA issues across the province of Ontario, Canada. A total of 840 caregivers were recruited.

**Results:**

259 participants (Mage = 45.94, SD = 7.11) identified as caregiving for at least one youth with MHA issues. The majority of the participants were female (70.7%), married (73.4%), and completed at least some college/Bachelor degree (59.1%). The mean age of youth was 16.72 years (SD = 5.33) and the most frequently reported diagnoses were Depression (30.1%), ADHD (27.8%) and Generalized Anxiety Disorder (21.2%). Regression results demonstrated that presently accessing services, presently seeking services, and higher levels of barriers MHA services were significantly predictive of identifying navigation as helpful for finding appropriate MHA services (χ^2^(7) = 28.69, *p* < .001, Nagelkerke R^2^ = .16). Furthermore, presently accessing services was significantly predictive of identifying case management as helpful (χ^2^(7) = 29.59, *p* < .001, Nagelkerke R^2^ = .156), and of identifying a primary healthcare provider as helpful (χ^2^(7) = 38.75, p < .001, Nagelkerke R^2^ = .197) for finding appropriate MHA services.

**Conclusion:**

Identifying the nature and extent of youth MHA issues, service needs, and family preferences can inform the development of services that address families’ needs and lend vital support for accessing services within a complex system.

## Background

Mental health and/or addictions (MHA) issues affect an estimated one in five Ontarians [1]. For approximately 70% of these individuals, the difficulties they experience can be traced back to childhood [2]. In Ontario, 467,000 to 654,000 children and youth experience at least one MHA concern at any given time [3], yet fewer than a third of those in need of care receive specialized or appropriate treatment [4, 5]. In Ontario, identified barriers to care for children and youth include, but are not limited to, ineffective transitions between types and levels of care, unclear mechanisms for accessing care, stigma, and the social determinants of health [6]. Even when youth do access MHA care, services are not optimal [7] and wait times for specialized treatment in Ontario can exceed one year [8]. These issues highlight the urgent need for facilitating access to needed care in this population.

Youth with MHA concerns present increased needs and demands on caregivers (including parents/guardians or other individuals in a primary caregiving role), resulting in lost productivity, stress, and consequences to employment [8, 9]. Families are often active in facilitating access to appropriate help, encouraging help-seeking behaviours in their youth, and are the source of important health-related information necessary for providers to complete accurate assessment and monitoring of outcomes [10, 11]. In fact, parental concern for their youth is often the initiating factor for referral to MHA services [10]. Caregivers often spend a great deal of time and effort seeking services for their youth, yet face numerous individual, social, and systemic barriers to finding needed services for their youth [11, 12]. Furthermore, youth with concurrent substance use and mental health concerns present even greater parental burden and place increased stress on the entire family [13]. It is, therefore, important to ensure families are able to access services for their youth early and effectively. However, little is known about families with youth with MHA concerns, their service use patterns, and preferences for support in accessing MHA services for their youth.

More recently, to enhance access to and transition through the MHA system of care for youth and their families, several organizations have developed system navigation services [14–17]. Despite the appeal and growth of navigation, little is known about the needs of families who support youth with MHA concerns with respect to access and transition throughout care as supplied by navigators, primary healthcare providers, case managers, peer support, and online and print self-help/information resources [10, 18, 19]. Thus, a community survey of caregivers of youth with MHA issues is warranted, to better understand their preferred methods of support in accessing services for their youth who are experiencing MHA concerns, across a range of options from more low intensity and passive methods (e.g., informational websites) to higher intensity and innovative models (e.g., case management, navigation).

### Aims of the study

The primary objective of this study was to identify and describe Ontario families who have a youth under age 30 with MHA issues and to explore their service needs. The secondary objective is to explore what family and youth factors are associated with families’ service/support preferences in accessing MHA supports for the youth and family.

## Methods

### Design

This was a cross-sectional community-based survey study in which Ontario adults caring for a youth up to age 30 with a MHA issue identified the issues the youth was experiencing, the burden presented to themselves and the youth, and their MHA service use, needs, and preferences. All study tools and methods were approved by the Sunnybrook Health Sciences Centre Research Ethics Board (485–2015).

### Research questions

1. What are the characteristics of families with a youth up to age 30 experiencing a MHA issue?
2. What are caregivers’ preferred methods of support for access to MHA services?
3. Are service barriers, service usage, and youth and caregiver difficulties associated with reported preferences for support in accessing MHA services?

### Data collection

#### Inclusion/exclusion criteria

The inclusion criteria for this study was adults between the ages of 35 and 65 (specified as such to increase likelihood that they would have a dependent youth), residing in the province of Ontario and with at least one dependent youth up to age 30. Participants who did not identify that they were caring for at least one youth with MHA concerns in the screening questions were excluded from continuing with the survey.

#### Survey data collection

A survey pilot to test acceptability was conducted with 5 participants who were caregivers of youth with MHA concerns and not involved in the development of the survey. There were no changes to survey content as a result of the pilot. Data collection was conducted in May–June, 2016. Recruitment was conducted through LightspeedGMI, a service that allows researchers to recruit its members based on target criteria, such as sociodemographic factors or daily behaviours, up to the desired sample size. LightspeedGMI sends online survey invitations to its members and offers respondents incentives that can be put toward charitable donations and sweepstake entries, limits the total number of surveys members can take per week, and regularly benchmarks their registrants to ensure that their respondent member demographics are representative of the general population. The survey was sent out to the 41,700 individuals available to be recruited through LightspeedGMI. There were a total of 840 responses from caregivers with and without youth with MHA concerns, indicating a response rate of 2%. LightspeedGMI typically receives response rates between 1 and 15%, depending on target sample eligibility. Participants were able to review study information prior to beginning the survey, and were provided with a toll-free number and email address to contact the study investigators with questions prior to participating.

### Survey tool

Data was collected by the research team using an online survey hosted on SurveyMonkey®, which consisted of 72 questions. All participants completed background questions and then, if applicable, responded to questions regarding MHA service use and service needs for a youth up to age 30 who was in their care and was experiencing a MHA concern. Those who identified more than one youth of concern were asked to respond to the survey focusing on the youth who presented the greatest concern. A copy of the full survey is available from the corresponding author upon reasonable request.

#### Sociodemographic background and screening (research question 1)

Participants were asked a series of sociodemographic background questions (e.g., age, gender, marital status) followed by a screening question that ascertained they were caring for at least one youth with an emotional, behavioural, or MHA issue of concern. See Table 1 for relevant questions and response items.

**Table 1:** Demographics of families with a youth with emotional/ behavioural/ addictions/ substance use concerns in Ontario

Background	Caregivers of youth with no MHA issue Mean (SD) or Count (% in group)	Caregivers of youth with MHA issue Mean (SD) or Count (% in group)
**Age in years**	47.1 (8.4)	45.9 (7.1)
**Gender**		
Female	365 (63.1)	183 (70.7)
Male	210 (36.3)	76 (29.3)
Transgender/Gender Non-Conforming	1 (0.2)	0 (0.0)
Unknown	5 (0.9)	0 (0.0)
**Marital Status**		
Married	423 (72.8)	190 (73.4)
Separated/Divorced	60 (10.3)	25 (9.7)
Civil Union/Cohabiting with Significant Other	58 (10.0)	28 (10.8)
Widowed	9 (1.6)	6 (2.3)
Single, never married	26 (4.5)	10 (3.9)
Unknown	5 (0.9)	0 (0.0)
**Education**		
High school or less	117 (20.1)	49 (18.9)
Some or complete bachelor	323 (55.6)	153 (59.1)
Graduate or professional degree	132 (22.7)	55 (21.2)
Unknown	9 (1.6)	2 (0.8)
**Area of Ontario**		
Greater Toronto Area	226 (38.9)	81 (31.3)
Southwestern Ontario	132 (22.7)	84 (32.4)
Eastern Ontario	92 (15.8)	37 (14.3)
City of Toronto	82 (14.1)	33 (12.7)
Northeastern Ontario	22 (3.8)	18 (6.9)
Northwestern Ontario	17 (2.9)	6 (2.3)
Unknown	10 (1.7)	0 (0.0)
**Community Size**		
Large Urban Area	352 (60.6)	154 (59.5)
Medium Population	113 (19.4)	50 (19.3)
Small Population	63 (10.8)	43 (16.6)
Rural Area	39 (6.7)	12 (4.6)
Unknown	14 (2.4)	0 (0)
**Number of Dependent Youth**	1.6 (0.96)	2.1 (0.99)

#### Youth mental health and addictions issues and needs (research question 1)

Those that screened positive were asked about the youth’s background (e.g. age, gender, caregiver’s relationship to the youth), mental health and behavioral issues, needs, professional diagnoses, and substances used (see Table 2).

**Table 2:** Prevalence of emotional/behavioural/addictions/substance use concerns in MHA caregiver sample

Background	Youth with MHA issue Mean (SD) or Count (% in group)
**Caregiver Relationship to Youth**	
Biological parent	217 (83.8)
Adoptive parent	18 (6.9)
Legal guardian	11 (4.2)
Relative (grandparent, aunt/uncle, cousin, sibling)	6 (2.3)
Step-parent	7 (2.7)
**Issues**	
Difficulties with academics	126 (49.6)
Outbursts of anger or rage	105 (41.3)
Difficulty sleeping	103 (40.6)
Lacking energy or motivation	100 (39.4)
Worrying constantly	98 (38.6)
Frequent or abnormal mood swings	97 (38.2)
Excessive technology use	96 (37.8)
Poor concentration or memory	85 (33.5)
Mean number of issues/youth	7.38 (5.3)
**Diagnoses**	
Depression	78 (30.1)
Attention Deficit Hyperactivity Disorder	72 (27.8)
Generalized Anxiety	55 (21.2)
Autism Spectrum Disorder	32 (12.4)
Obsessive Compulsive Disorder	21 (8.1)
Mean number of diagnoses/youth	1.40 (1.14)
**Diagnosis Type**	
Mental Health Diagnosis only	99 (38.2)
Behavioural Diagnosis only	93 (35.9)
None	52 (20.1)
Concurrent (Mental Health and Addiction)	10 (3.9)
Behavioural and Addiction	3 (1.2)
Dual Diagnosis	2 (0.8)
Addiction Only	0 (0)
**Substances**	
None	161 (62.2)
Cannabinoids	55 (21.2)
Cigarettes/Nicotine	48 (18.5)
Alcohol	38 (14.7)
Prescription Narcotics	11 (4.2)
Stimulants	10 (3.9)

#### Services (research question 2 and 3)

Lists of services were compiled for the purposes of this survey, and included general service categories (e.g., treatment from a psychiatrist, treatment from a psychologist, treatment from a family doctor, counselling, residential treatment, educational/vocational supports, case management; see Supplement Table 2). Respondents were asked to indicate which services, if any, they were currently accessing and which they were currently seeking for their youth’s MHA care, and to rate their general satisfaction with services accessed presently and in the past.

To assess preferences for service delivery and methods, participants were asked to rate the perceived helpfulness of various service providers, based on the functions of these roles as well as the perceived importance of different types of support in facilitating access to MHA resources for the youth and family. These options were phrased in terms of the type of support provided rather than using a specific term for the service provider (e.g., “a professional who coordinates services in the community for this youth” rather than “case manager”) to lend consistency to participants’ understanding of the roles. Participants were also asked to indicate their preferences for various modalities of service provision (e.g., varying lengths of involvement, in-person vs. online). These service options and modalities of provision were also developed with the consulting team. See Table 3.

**Table 3:** Service preferences

Service types endorsed as helpful for finding appropriate mental health service for the youth with mental health and/or addictions concerns	n(%)	
Navigation	201 (77.6)	
Case Management	188 (72.6)	
Primary Health Care Provider	182 (70.3)	
Peer Support for Caregiver	178 (68.7)	
Peer support for Youth	156 (60.2)	
Self-Help Resources	132 (51.0)	
**Support types endorsed as important for help finding mental health and/or addictions resources for the youth and family**	n(%)	
Supporting in connecting with services	228 (88.0)	
Specific suggestions based on matching	222 (85.7)	
General guidance on the system	216 (83.4)	
List of services in community	216 (83.4)	
Support for other community services (e.g., housing, employment)	169 (65.3)	
List of services in province	166 (64.1)	
**Preferred length of involvement of service supporting connection to mental health and/or addictions resources**	n(%)	
As long as it takes to find the right services	114 (44)	
Remains involved as needed even after connecting	74 (28.6)	
For a few weeks	35 (13.5)	
For a few months	18 (6.9)	
One time contact	9 (3.5)	
**Preferred mode of communication with service supporting connection to mental health and/or addictions resources**	**Mean Rank (1 = least preferred)**	**SD**
Face to face/in-person	6.72	1.80
Over the phone	6.35	1.67
Via Email	5.65	1.68
Written materials (i.e., fliers, pamphlets)	4.43	1.76
Websites, online publications	4.00	1.73
Regular mail	3.77	1.68
Text messaging	3.60	1.97
Social media	2.40	1.87

#### Youth burden (research question 3)

To identify the level of disruption experienced by the youth as a result of their MHA issues, items pertaining to youth burden that frequently present in this population (e.g., social relationships, school/work) were developed by a consulting team of social workers, counsellors, and psychiatrists with experience working with youth and families in need of MHA care. This team also suggested wording to ensure clarity. See Supplement Table 1 for the full list of items.

#### Caregiver burden (research question 3)

To identify the level of disruption presented to the caregiver as a result of the youth’s MHA issues, questions pertaining to caregiver burden were developed by selecting and revising relevant items from a known scale (i.e., Caregiver Strain Questionnaire Short Form [20]) and by the consulting team. This team suggested the addition of items regarding disruption of social relationships and caregiver’s physical health. See Supplement Table 1 for the full list of items.

#### Barriers (research question 3)

Questions pertaining to barriers to care were developed based on the known literature on barriers to MHA service access for families [21] and by the consulting team. Participants were asked to rate the extent to which various factors posed a barrier to service. See Supplement Table 1 for the full list of items.

### Analysis

Descriptive variables were calculated for all sociodemographic items for the caregivers and youth. Chi-square analyses were conducted for each sociodemographic factor to determine whether there were any significant differences between caregivers of youth with MHA concerns as compared with caregivers of youth without these concerns. The most frequent responses for certain variables were identified and used to dichotomize the variable (e.g., Marital Status: “married” (most frequent response) vs. “not married” (all other responses). Finally, in cases where every category needed to be considered (i.e. Ontario Area), separate 2 × 2 chi-squares were conducted (i.e., Southwestern Ontario vs. all, Eastern Ontario vs. all, etc.) with Bonferroni correction.

To explore predictors of families’ service preferences, multiple logistic regression was performed for each of the service types most frequently endorsed as potentially helpful to families. Variables pertaining to youth and caregiver characteristics (i.e., youth burden, caregiver burden, number of presenting MHA issues) and service use (i.e, barriers, accessing services, seeking services, and an interaction between accessing and seeking services) were entered into each model as predictors of each of the top three services that caregivers identified as helpful for connecting with appropriate MHA care for their youth (i.e., navigation, case management, and family physician). Navigation service was defined as “clinically trained health professional who would assess the youth and family’s needs, explore treatment options, connect them with appropriate service matches for the youth, and provide support for the whole family.” Case Management was defined as previously described. A primary healthcare provider was defined as a family doctor or pediatrician. The internal consistency of related survey items (i.e. scales entered into the model as predictors of perceived helpfulness) were assessed with Cronbach’s alpha and through confirmatory factor analysis.

## Results

### Sociodemographics

All sociodemographic information for both those who identified as a caregiver of a youth with a MHA concern and those who were caregivers of youth without these concerns is displayed in Table 1. There was no significant association between group and marital status (χ ^*2*^ (1) = .001, *p* = .981), education level (χ^2^ (1) = .285, *p* = .593), community size χ^2^ (1) = .515, *p* = .473), living in Northern Ontario (χ^2^ (1) =1.508, *p* = .219), living in Eastern Ontario (χ^2^ (1) = .453, *p* = .501), or living in the City of Toronto (χ^2^ (1) = .392, *p* = .532. There was a significant difference between groups in caregiver age (t (577) = − 2.046, *p* = .041), in that caregivers of youth with MHA concerns (m = 45.9, SD = 7.1) were younger than respondents who were not caring for youth with MHA concerns (m = 47.1, SD = 8.4). There was also a significant difference between groups in the number of any dependent youth (t(835) < .001), in that caregivers of youth with MHA concerns (m = 2.1, SD = .999) were caring for more dependent youth than respondents who were not caring for youth with MHA concerns (m = 1.6, SD = .962). There was a significant association between group and gender (χ^2^ (2) = 4.54, *p* = .104); that is, the proportion of female respondents was higher in caregivers of youth with MHA than in those with youth with no MHA concerns. There was also an association between group and geography, in that the proportion of caregivers of youth with MHA in the Greater Toronto Area Toronto Area in the sample was significantly lower than expected (χ^2^ (1) = 5.274, *p* = .022) and the number of caregivers living in Southwestern Ontario was significantly greater than expected (χ^2^ (1) = 8.031, *p* = .005), indicating slight under- and over-representation of these populations, respectively.

### Mental health and/or addictions issues

A total of 259 (30.1%) respondents indicated that they were caring for at least one youth experiencing an emotional, behavioural, mental health, or addictions issue of concern (see Table 2). The most frequently reported diagnoses are displayed in Table 2. Approximately 81% of the sample reported having received a diagnosis for their youth. Depression was the most commonly reported diagnosis in the youth, and was indicated in approximately 30% of the sample. Among those reporting any substance use for their youth, the most frequently reported substance used was cannabis (21.2%; see Table 2).

### Caregiver and youth burden

The measure of caregiver’s perception of youth burden was highly internally reliable (Cronbach’s α = .80). The most prevalent source of strain for youth, as perceived by caregivers, was the youth’s social relationships (M = 2.95, SD = 1.2). The measure of caregiver’s own burden was also found to be highly internally reliable (Cronbach’s α = .91). The most common source of burden for caregivers was feeling tired or strained as a result of the youth’s MHA issues (M = 3.3, SD = 1.2; see Supplement Table 1).

### Service Use Patterns

The measure of barriers to service was highly internally reliable (Cronbach’s α = .86). The most prevalent barrier identified by caregivers was the youth’s (lack of) motivation to participate in service (m = 3.1, SD = 1.7; see Supplement Table 1). A total of 60.2% (*n* = 156) of participants identified that they were currently accessing services for their youth. The most commonly accessed services were treatment from a physician (37.8%), treatment from a psychiatrist (18.1%), and treatment from a psychologist (16.6%). However, of those currently accessing services, 51.1% were not satisfied with the services they were receiving. Of those who were currently accessing services, 66% (*n* = 103) identified that they were also currently seeking service. Overall, 64.5% (*n* = 167) of the entire sample were currently seeking services whereas 15.1% (*n* = 39) were neither accessing nor seeking services. The most commonly sought services were treatment from a psychiatrist (19.7%), general assessment for MH concern (19.3%), and treatment from a psychologist (18.9%) (see Supplement Table 2). Of note, 48.9% of participants were not satisfied with services they had utilized in the past. Among families who were involved with more than one service, 42.7% were not satisfied with the collaboration (i.e., communication and connections) between services. Finally, 21.2% of participants indicated that they were currently on a wait list for service. Of these, 54.5% had already been waiting over six months for service. Of those who had already been waiting this time, 70% expected they would be waiting at least another six months.

### Service preferences

The most commonly endorsed preferred services were family navigator (77.6%), case manager (72.6%), primary health care provider (70.3%). More passive forms of support, such as self-help books (51.0%), were less frequently endorsed. When asked to rate the professional support types caregivers thought would be most important in facilitating access to services, the supports most commonly endorsed were: support in connecting with services (88.0%), specific suggestions for MHA services based on careful matching of youth and family needs to the expertise of a service provider (85.7%), a list of MHA resources in the community (83.4%), general guidance on how the MHA system works (83.4%), support for other “non-treatment” community services like housing and employment (65.5%), and a list of MHA resources in the community and across the province (64.1%; see Table 3).

Delivery formats for services that would support access to the most appropriate services for the youth and family were also explored (Table 3). Face-to-face was ranked the highest, on average (M = 6.7, SD = 1.8), followed by phone (M = 6.4, SD = 1.7)), and email (M = 5.6, SD = 1.7). Rankings ranged from 1 to 8, with a ranking of 8 indicating highest preference. Regarding length of involvement, the majority of participants preferred the professional service coordinator stay involved for as long as it takes to find the right services (44.0%), followed by remaining involved as needed even after connecting to the right services (28.6%), or for a few weeks (13.5%; see Table 3).

### Predictors of perceived helpfulness of services

To address the third sub-question, individual logistic regressions were conducted of each of the top three service types most frequently endorsed as helpful for accessing MHA resources (i.e., navigation, case management, primary healthcare provider.) The predictor variables were difficulties experienced (number of emotional, behavioural, mental health, and/or substance use issues displayed by the youth, caregiver burden, and youth burden) and service use histories (accessing service, seeking service, barriers to service) reported by caregivers (see Table 4).

**Table 4:** Multiple Logistic Regressions of Perceived Helpfulness of Supports for Connecting to MHA Resources

	Navigation				Case Management				Physician			
	beta	p	OR	95% CI	beta	p	OR	95% CI	beta	p	OR	95%CI
Accessing Services	1.42	.004*	4.11	1.58–10.90	1.26	.007*	3.53	1.42–8.79	2.20	.001*	9.02	3.28–24.86
Seeking Services	1.05	.030*	2.87	1.10–7.43	.72	.113	2.05	.843–5.00	.76	.095	2.14	.88–5.22
Accessing xSeeking	−1.26	.052	.283	.08–1.01	−.40	.509	.67	.21–2.19	−.97	.121	.38	.11–1.29
Barriers	.06	.007*	1.06	1.02–1.10	.01	.746	1.01	.97–1.05	−.02	.246	.98	.94–1.02
Caregiver Burden	.01	.796	1.01	.95–1.07	.04	.136	1.04	.99–1.10	.04	.147	1.04	.99–1.10
Youth Burden	−.05	.301	.95	.86–1.05	.09	.87	1.09	.92–1.11	−.004	.939	.99	.91–1.09
Behaviour Issues	.02	.537	1.02	.95–1.10	−.02	.556	.98	.91–1.05	−.01	.805	.99	.93–1.06
Full Model	χ^2^(7) = 28.69, p < .001, Nagelkerke R^2^ = .16				χ^2^(7) = 29.59, p < .001, Nagelkerke R^2^ = .156				χ^2^(7) = 38.75, p < .001, Nagelkerke R^2^ = .197			

* denotes significance at the .05 level

The logistic regression model of the effects of difficulties experienced and service use histories on the likelihood of identifying navigation service as helpful for accessing MHA resources was significant (χ^2^(7) = 28.69, *p* < .001). The model explained 16.0% (Nagelkerke R^2^) of the variance in selecting navigation as helpful and correctly classified 81.1% of cases. Variables that independently and significantly predicted variance in preference for navigation were: level of barriers experienced (*p* = .007, OR = 1.06, 95%CI = 1.02–1.10), whether the family was currently accessing services for MHA support for the youth (*p* = .004, OR = 4.14, 95%CI = 1.58–10.90), and whether the family was currently seeking services for MHA support for the youth (*p* = .030, OR = 2.87, 95%CI = 1.10–7.43).

The logistic regression model predicting likelihood of identifying case management service as helpful for accessing MHA resources, based on difficulties experienced and service use histories was significant ((χ^2^(7) = 29.59, *p* < .001, Nagelkerke R^2^ = .156). The model correctly classified 74.5% of cases. The only variable that significantly predicted variance in perceived helpfulness of case management was whether the family was currently accessing services for MHA support for the youth (*p* = .007, OR = 3.532, 95%CI = 1.42–8.49) .

The logistic regression predicting likelihood of identifying a primary healthcare provider as helpful for accessing MHA resources, based on difficulties experienced and service use histories was also significant ((χ^2^(7) = 38.75, *p* < .001, Nagelkerke R^2^ = .197). The model correctly classified 74.1% of cases. The only variable that significantly predicted variance in perceived helpfulness of a primary healthcare provider was whether the family was currently accessing services for MHA support for the youth (p < .001, OR = 9.023, 95%CI = 3.275–24.858).

## Discussion

This study demonstrates that approximately 30% of Ontario families with youth under 30 are providing support for at least one youth with a MHA concern. Moreover, these families most strongly endorse navigation services, followed by case management and support from a primary care provider as preferred ways to find and access MHA services for their youth. In terms of length of involvement, caregivers indicated the strongest preference for a service coordinator that would stay involved as long as needed to find the right services followed by a preference for a service coordinator who would remain involved as needed after connecting the youth to services. Of the possible modes of support, caregivers’ strongest preference was for face-to-face contact, followed by phone then email contact. MHA issues in Ontario youth have previously been studied and have reported a similar profile in terms of the types and rates of issues experienced by the youth in the current study [22–24]. However, these concerns are less frequently explored from the perspectives of caregivers who may bear significant responsibility for the well-being of the youth. Caregivers have considerable insight into the issues and concerns of the youth as well as the supports needed to connect with the most appropriate care [10, 25]. Prior work has explored the caregiver’s needs and level of burden [26] and difficulties associated with obtaining services [11], but has not explored service preferences. Slaunwhite and colleagues [26] identified emotional support and help from medical professionals as key needs in the caregiving of individuals with mental health concerns in a Canadian sample. Caregiving for mental health concerns was identified as presenting significantly greater burden than other caregiving roles, including physical and other diseases [26], thus emphasizing the importance of exploring and understanding the caregiver perspective regarding youth MHA issues.

The current study found a substantial proportion of participants experienced barriers to service access and service needs, wait times in excess of 6 months, and dissatisfaction with previous services. These findings point to areas of concern in the healthcare system, some of which have been suggested previously in Ontario and beyond [27]. Navigation and case management have been shown to help individuals with mental health concerns feel supported in finding and accessing services [28, 29], and thus have potential to help families and youth connect to the most appropriate type and level of care for their needs. Navigation and case management resources help families move towards the care they need for their youth, and therefore further investment in these types of services may be worth exploring. However, there are few controlled trials to date of navigation in the MHA system and more evidence is needed on the cost-benefit of this intervention before widespread adoption of this model can be supported.

This study demonstrated that certain family and youth features significantly and independently predict whether or not navigation is identified as potentially helpful: 1. whether or not a family was currently accessing services, 2. whether or not a family was currently seeking services, and 3. the level of barriers to service experienced. The finding that preference for navigation is predicted by seeking services makes intuitive sense; as navigation is geared at helping patients and families access and transition through the system [30], it is appropriate and may be helpful for those that are looking for support. Those who are currently accessing services may also understand the value of support in connecting with appropriate services and the desire for these families for navigation may be influenced by dissatisfaction with previous services, as observed in the present sample. Navigation may therefore be appropriate for families at any stage of their journey through the MHA system. Furthermore, another central goal of navigation is overcoming barriers [30]. The description provided to subjects regarding navigation may have resonated with their desire to alleviate barriers to care for their youth with MHA issues. Finally, it is also possible that some respondents had experienced elements of navigation previously, either from a specific service or as a function of prior or current care teams, which may also have resonated positively or negatively and influenced their perceptions of this mode of support.

The variable “Currently accessing services” was significantly predictive of perceiving case management as helpful, regardless of the level of need in other areas or whether caregivers were seeking services. Families that are connected to services may still experience difficulty managing the requirements of participating in care and see the value of case management as a service that would support these activities. Accessing services was also significantly predictive of identifying support from a primary healthcare provider, such as a family doctor, as helpful. Family doctors are often viewed by patients as a central point of contact and triage for mental health concerns [10], yet family doctors may be unable to support the range of youth MHA needs themselves due to several reasons including a lack of time within appointments coupled with a need for further training specific to mental health [31]. Thus, specialized supports with knowledge and expertise in youth MHA may act as a valuable complement in assisting families with coordinating care traversing the MHA system.

Caregiver preferences for their youth’s treatment are an important determining factor in youth involvement with services [32]. Although prior work focused on attendance at treatment sessions [32], there may be similar implications for families accessing services for youth. In a system where youth are often described as falling through cracks during transitions [33], having consistent follow-up to ensure the youth and family are well-connected is valuable. Face-to-face contact was the most preferred mode of contact, followed by email and phone. While face-to-face connections are valuable in MHA services, more “virtual” types of youth MHA and family supports such as phone and email are becoming popular, especially since there may be time constraints, multiple appointments to coordinate, and/or inconvenient location of service [34]. Text and social media were among the least preferred methods of communication in this sample of caregivers. Such web-based supports, in particular, have become increasingly important to youth, due to the pervasiveness of online communication in their everyday lives [34]. Also important to consider is the ability of web-based supports to reach youth and families who are not located near the service provider, to improve access to care in general and in a cost-effective manner [34]. While there is considerable potential for virtual supports like telepsychiatry to help patients overcome barriers to care, existing evidence indicates it is not as pervasive as it can be, and is underutilized in the areas of Ontario where patients may benefit from such access to care [35]. However, there is a great deal of potential for evolution in this system, as these types of supports can also be incredibly valuable in times where physical distancing measures limit the ability of providers to meet in-person with clients [36]. For services aimed at supporting both youth and families, the impact of potential incongruities in preference warrant further exploration to ensure the design of services that meet both youth and family expectations.

Of note was also the small subset (*n* = 39; 15.1%) of participants who had identified existing MHA issues in their youth and were neither accessing nor seeking services. There are many reasons youth and families avoid care or become disconnected from the system, including stigma, the youth’s motivation, ineffective transitions in care, previous negative experiences with services, or a disinterest or ambivalence toward the need for service [10, 25, 37, 38]. A dedicated study is needed to understand what keeps these youth and families outside of the system, and whether there are existing resources that can help them become connected to supports if needed.

There are limitations to this work. First, recruitment through a market research firm limited knowledge about the participant sampling frame. Although there is regular benchmarking of respondent demographics and targets by the firm, the true representativeness of the sampling frame for the population of Ontario could not be known. However, this recruitment strategy afforded broad access to respondents within the financial and time constraints of this study. Furthermore, comparisons of key respondent demographics to that of the population of province in general were determined to be within acceptable margins (i.e., no greater than a 10% difference between the survey sample and general population on key demographic frequencies, such as gender and marital status). Although the sample was representative in general, greater representation from male caregivers, single female caregivers, or from more caregivers who had not completed higher education would have allowed for greater generalizability to these groups. Second, associated with recruitment via market research companies is the potential for participants to hastily complete surveys for credit. To limit this possibility, LightspeedGMI panel respondents were limited in the number and frequency of surveys that could be completed. Screening questions were carefully set so inclusion/exclusion criteria could be ascertained and only those with relevant experience could participate. Furthermore, regular data cleaning practices (e.g., removal of anomalous cases, creation of new response categories for repetitive text responses) were applied to the dataset to further reduce the possibility of misrepresentative data being entered into the final analysis. Additionally, it is possible that there were additional categorizations/types of services that participants may have been accessing or seeking for their youth, which were not represented. Future work may consider the addition of open-text responses and/or exploring qualitatively participants’ experiences of accessing or seeking services in relation to the service types sought after by participants. Finally, although study or investigator affiliations with existing navigation services were not presented in the body of survey, these were mentioned in the informed consent documentation presented to participants. This might have inadvertently influenced respondents to be more positively disposed towards navigation. To mitigate potential for such response bias, service preferences were queried primarily in terms of the roles and functions of the service provider rather than the name of the service type.

## Conclusions

Overall, the findings of this study highlight that a large proportion of Ontario families are caring for youth with MHA concerns. Although the MHA system contains numerous resources, youth and families face difficulties accessing needed care and desire support with finding the most appropriate care for their needs. These findings highlight the importance of innovative system solutions that involve direct contact with a professional to guide the family and youth with access to and transition through the care system.

## Supplementary information

**Additional file 1: Supplement Table 1**. Youth Burden, Caregiver Burden, and Barriers to accessing MHA services.

**Additional file 2: Supplement Table 2**. Services Presently Accessing or Seeking.

## Data Availability

The datasets used and/or analysed during the current study are available from the corresponding author on reasonable request.

## Abbreviation

MHA: Mental health and/or addictions
