# Supplementary material for: The needs and service preferences of caregivers of youth with mental health and/or addictions concerns
Source: BMC Psychiatry. 2020 Aug 14;20:409. doi: 10.1186/s12888-020-02801-y (PMC7427896; doi:10.1186/s12888-020-02801-y)
Supplement: Supplementary file 1 — Additional file 1: Supplement Table 1. Youth Burden, Caregiver Burden, and Barriers to accessing MHA services. [file 12888_2020_2801_MOESM1_ESM.docx]

Supplement Table 1. Youth Burden, Caregiver Burden, and Barriers to accessing MHA services

| **Sources of Youth Burden** | **M** | **SD** |
| --- | --- | --- |
| Social Relationships | 2.95 | 1.17 |
| Schooling/Work | 2.88 | 1.32 |
| Family Relationships | 2.69 | 1.09 |
| Leisure/recreational activities | 2.63 | 1.26 |
| Risk for harm to self or others | 1.86 | 1.31 |
| Youth Burden Total Score | 13.02 | 4.58 |
| **Sources of Caregiver Burden** | **M** | **SD** |
| Worried about youth’s future | 3.88 | 1.05 |
| Tired or strained as a result of youth’s issues | 3.34 | 1.22 |
| Unhappy as a result of youth’s issues | 3.25 | 1.16 |
| Personal time interrupted | 2.92 | 1.13 |
| Negative impact on family relationships | 2.88 | 1.18 |
| Negative impact on general health | 2.73 | 1.27 |
| Negative impact on social relationships outside the family | 2.38 | 1.28 |
| Financial strain | 2.31 | 1.42 |
| Missed work or other duties to support the youth | 2.06 | 1.32 |
| Caregiver Burden Total Score | 25.76 | 8.38 |
| **Barriers** | **M** | **SD** |
| Youth’s motivation to participate in mental health care | 3.12 | 1.74 |
| Most appropriate treatment options are not readily available | 2.71 | 1.66 |
| Costs of services | 2.65 | 1.84 |
| Youth’s knowledge about the mental health and addictions care system | 2.50 | 1.70 |
| Caregiver’s lack of knowledge about the mental health and addictions care system | 2.47 | 1.56 |
| Confidentiality issues (i.e., unable to discuss this youth’s personal health information with the people involved in care without the youth’s consent) | 2.31 | 1.80 |
| Youth’s geographical location | 1.99 | 1.65 |
| Availability of culturally-sensitive services | 1.49 | 1.62 |
| Barriers Total Score | 19.26 | 9.65 |
