# Supplementary material for: The needs and service preferences of caregivers of youth with mental health and/or addictions concerns
Source: BMC Psychiatry. 2020 Aug 14;20:409. doi: 10.1186/s12888-020-02801-y (PMC7427896; doi:10.1186/s12888-020-02801-y)
Supplement: Supplementary file 2 — Additional file 2: Supplement Table 2. Services Presently Accessing or Seeking. [file 12888_2020_2801_MOESM2_ESM.docx]

Supplement Table 2. Services Presently Accessing or Seeking

| **Services** | **n(%) accessing** | **n(%) seeking** |
| --- | --- | --- |
| Treatment from a family physician/walk-in clinic doctor | 98 (37.8) | 19 (7.3) |
| Treatment from a psychiatrist | 47 (18.1) | 51 (19.7) |
| Treatment from another MH professional | 45 (17.4) | 48 (18.5) |
| Treatment from a psychologist | 43 (16.6) | 49 (18.9) |
| Assessment for a mental health concern | n/a | 50 (19.3) |
| Financial support | 34 (13.1) | 34 (13.1) |
| Educational/vocational supports | 31 (12.0) | 27 (10.4) |
| Parent/caregiver support | 28 (10.8) | 44 (17.0) |
| Case management | 20 (7.7) | 25 (9.7) |
| Crisis support/intervention | 15 (5.8) | 14 (5.4) |
| Addiction assessment/treatment | 10 (3.9) | 17 (6.6) |
| Out-patient hospital treatment program | 7 (2.7) | 15 (5.8) |
| Supportive housing | 5 (1.9) | 11 (4.2) |
| In-patient hospital treatment program | 3 (1.2) | 6 (2.3) |
| Residential treatment facility | 2 (.8) | 10 (3.9) |
| Out of province/out of country services | 2 (.8) | 4 (1.5) |
| Lesbian, Gay, Bisexual, Transsexual, Queer/2 spirit supports | 1(.4) | 5(1.9) |
| Culturally specific services | 0 | 6 (2.3) |
